# Communities’ knowledge, perceptions and preventive practices on soil-transmitted helminthes in Jimma, Oromia, Ethiopia: Formative mixed study

**DOI:** 10.1371/journal.pntd.0012483

**Published:** 2024-09-20

**Authors:** Daba Abdissa, Yohannes Kebede, Morankar Sudhakar, Gelila Abraham, Gebeyehu Bulcha, Teshome Shiferaw, Nimona Berhanu, Firanbon Teshome, Hirpa Miecha, Zewdie Birhanu

**Affiliations:** 1 Department of Biomedical Sciences, Jimma University, Jimma, Ethiopia; 2 Department of Health, Behavior and Society, Jimma University, Jimma, Ethiopia; 3 Department of Health Policy and Management, Jimma University, Jimma, Ethiopia; 4 Jimma Zone Health office, Oromia, Ethiopia; 5 School of Pharmacy, Jimma University, Jimma, Ethiopia; 6 Oromia, regional health bureau, Oromia, Ethiopia; Federal University of Agriculture Abeokuta, NIGERIA

## Abstract

**Background:**

Soil-transmitted helminthes (STH) infections are one of the most common neglected tropical diseases. It has become one of a significant public health problem programmatically aimed for prevention and control in Ethiopia. Limited evidence is available on communities’ knowledge, perceptions, and practices regarding STH particularly in rural settings of Jimma, Ethiopia.

**Methods:**

A community-based cross-sectional study triangulated with the qualitative method was conducted. The survey included 732 sampled rural households. Linear regression was used to assess association between predictors of knowledge and preventive practices of STH; likewise logistic regression was used to identify the predictors of hand washing practice at critical times. Kruskal-Wallis and Mann-Whitney tests were done to test differences in median risk perception score by socio-demographic factors. Qualitative data were collected through 7 key informant interviews, 6 focus group discussions and 7 expert group discussions then transcribed verbatim. Then, the data were coded, categorized and thematized using the Atlas ti.7.1.4 software package.

**Results:**

Almost all of the respondents (99.6%) had heard of STH. The prevalence of comprehensive knowledge, risk perception and preventive practices towards STH were 46.7%:(95%CI:43.2, 50.4), 55.2%: (95%CI:51.2,59) and 44.4%:(95%CI:40.8, 48.2) respectively. Likewise, the magnitude of knowledge and practice of hand washing at critical times were 42.5%: (95%CI: 38.7,45.9) and 43.9%: (95%CI: 40, 47.5) respectively. Risk perception and comprehensive knowledge towards STH varied significantly across districts and by respondents’ educational status. Ownership of improved latrine was associated to comprehensive knowledge of STH. The STH preventive practice that varied across districts was predicted by the overall and knowledge specific to washing hands at critical times. The practice of washing hands at critical times was significantly associated to knowledge of hand washing, owning improved latrine, and age from 15 to 34 year compared to >45 year. Moreover, qualitative findings were supportive of the findings.

**Conclusion:**

Despite reported exposures to STH communication opportunities, the study found modest levels of knowledge, perceptions, and preventive practices related to STH among rural communities where the burden of STH was the programmatic concern. These levels of knowledge, perceptions, and practices varied across the districts. Educational and latrine status predicted overall knowledge, whereas knowledge specific to hand washing and overall knowledge were predictors of STH preventive practice. Furthermore, washing hands during critical times was moderately improved among the young-aged, ownership of improved latrine and knowledgeable on hand washing. This study underscores the need for locally tailored and contextualized community behavioral change interventions needs to be strengthened toward improved STH preventive practices.

## Author summary

Soil-transmitted helminthes (STH) infections are one of the most common neglected tropical diseases and a significant public health problem in developing countries, despite numerous efforts to combat it. One effective yet short-term control method is preventive chemotherapy. A more comprehensive program of improved water, sanitation, and hygiene (WASH), along with knowledge of and use of its preventive measures, are needed for its long-term control and eventual elimination. This study examined community knowledge, perceptions and preventive practices towards STH using mixed method in Jimma, Oromia, Ethiopia. The finding revealed that, despite the reported exposures to STH communication opportunities, there were modest amount of knowledge, perception and preventive practices among communities and it varied across districts. Educational and latrine status predicted overall knowledge, whereas knowledge specific to hand washing and overall knowledge were predictors of STH preventive practice. Furthermore, washing hands during critical times was moderately improved among the young-aged, having improved latrine and knowledgeable on hand washing. This study underscores the need for local tailored and contextualized community behavioral change interventions.

## Background

Soil transmitted helminthes (STHs) are a group of parasitic intestinal worms which are the most common Neglected Tropical Diseases (NTDs)in sub-Saharan Africa[1],[2]. *Ascaris lumbricoides*, *Trichuris trichiura* and hookworms (*Necator americanus* and *Ancylostoma duodenale*) are the three main species of concern that infect humans [3],[4]. These parasites are transmitted by eggs shed in human faeces which contaminate the environment in areas with poor hygiene and sanitation. *Ascaris lumbricoides* and *Trichuris trichiura* are transmitted through the ingestion of infective eggs [5]. Both hookworm species and *Strongyloides stercoralis* infect human through larvae penetrating the skin, but *Ancylostoma duodenale* can also spread through consumption of larvae [6].

Despite various efforts to combat STH infections (STHI), it continues to pose a major socio-economic challenge in people living in, or coming from, areas with poor access to improved water, sanitation and hygiene(WASH)[7],[8]. The Global Burden of Diseases 2016 report shows that STHIs affect 1.5 billion people globally [9]. STH is one of the main public health issues affecting over 79 million people in Ethiopia [10–12]. It is endemic in 89% of the districts of the country [13] and its prevalence ranges from 18.1% to 70.3% [14–17].

The vulnerable groups of STHI are pregnant women, children in school, adults in high-risk occupations, and preschool-aged children [10],[18]. STHI are influenced by various factors such as socioeconomic, behavioral, environmental, and biological factors [19]. Low socioeconomic status, poor sanitation, low awareness, climatic conditions, educational status, risky occupations such as night soil reuse and farming [20], poor personal hygiene and the lack of access to safe drinking water are some of the major factors contributing to an increased risk of its infection, transmission and associated morbidity and mortality [21–23]. The majority of Ethiopia, including the study’s geographical area, has annual mean temperatures that are conducive to the spread of STHIs [24].

STHIs are responsible for school absenteeism, delayed physical growth, impaired cognitive development [25], small bowel or bile duct obstruction [26], malnutrition, anemia, educational loss, and poor pregnancy outcomes [27]. Furthermore, the devastating effects of STHIs could reduce economic output and keep endemic communities trapped in a cycle of poverty [28].

To prevent and control STHIs, the World Health Organization (WHO) recommended a periodic preventive chemotherapy (PC) as mass drug administration (MDA) with promotion of WASH and effective social and behavioral change communication (SBCC) interventions [29]. Ethiopia has started a national MDA to control STHs for children aged 5 to 19 and, starting in 2022, women who are of reproductive age in accordance with the WHO strategic plan. Deworming, however, only has a temporary impact on transmission, cannot stop reinfection, and does not kill immature worms. As a result, rapid human reinfection follows a successful PC [30].

Even where there are continuous control programmes, there is evidence of declining uptake of drug due to fear of treatment, insufficient MDA coverage [31], focused on specific risk groups, poor communication and logistics problems [31–33]. On the other hand, adults are more likely to contract the disease, which serves as a reservoir for its spread and evidences suggests that in order to stop the spread of STH, other at-risk groups in the community must also be included in order to maximize the effectiveness of interventions [34–36]. The persistence of STHI is attributed to a number of factors, including poorly coordinated interventions, a failure to integrate PC related interventions with other interventions like WASH, and a lack of emphasis on community knowledge[37], [38].

One essential and very successful strategy to lower exposure to and transmission of STHI is to promote WASH practices and access, which will allow for long-term, efficient control and eventual elimination of the infection [39], [40]. But over 25% of the world’s population suffers from STHIs, primarily as a result of poor access to sanitation [41]. According to a 2019 Ethiopian mini-demographic survey, only 9.7% and 61% of rural Ethiopian households, respectively, had access to improved water sources and improved sanitation facilities [42]. Moreover, knowledge and services of WASH are inadequate [43–45]. For instance, a study done in Eastern Ethiopia, indicated that 48.3% of the respondents think latrines are only intended for rich people [45].

SBCC interventions are inexpensive, efficient, and crucial for behavior change when combined with other interventions, especially for disadvantaged rural communities [46],[47]. According to studies, perceptions, knowledge, and preventative practices of STH on both an individual and community level are crucial factors for sustained control and eventual elimination of STHI [48],[49]. Furthermore, knowledge and appropriate perception of a disease are important for implementing preventive measures and health service utilization [50],[51]. Re-infection is common due to a combination of all people not receiving the drug and high exposure to the parasites through inadequate access to clean water and sanitation, making these infections challenging to eradicate. Improving the community’s perception and understanding of the recommended preventive methods is essential. However, in Ethiopia, where access to and utilization of the WASH program are inadequate, there is a dearth of evidence regarding community knowledge, perception, and preventive practices regarding STH and the majority of studies focus primarily on school-age children [11].

Therefore, this study was carried out to determine knowledge, perceptions and preventive practice towards STH using mixed method approaches in Jimma, Oromia, Ethiopia to provide an enhanced and comprehensive response to the problem thereby contributing to evidence-based STHI interventions. Moreover, water, sanitation status and hygiene practice were assessed which are critical in STH control. The results would be useful in creating community education and awareness raising interventions to support ongoing efforts to prevent, control and eliminate STH in Ethiopia and other similar settings.

## Methods and materials

### Ethical statement

The study protocol was received and approved by an ethics review committee of the Institute of Health, Jimma University (ref No: JHRPGD/344/2021). The purpose of the study was explained to each respondent and each participant received detailed information about the study, benefits, and risks of participation. In order to participate in the study, all adults (age ≥18) were signed a written consent form. For participants under the age of 18, written parental consent was taken from the parent (guardian) in accordance with the Declaration of Helsinki. The privacy and confidentiality of the information were ensured.

### Study setting and design

A community-based cross-sectional study supported by qualitative study was conducted in five rural districts (Gomma, Manna, Kersa, Omo Neda, and Omo Beyam) of Jimma zone, Oromia, Ethiopia from October-November 2021. Jimma zone is located 350 km away from Addis Ababa, the capital city of the country. The districts were identified through discussions with the zonal and regional health NTD experts considering key criteria such as the high burden of STH. From each district, two Gandas (lowest administrative unit in Oromia, Ethiopia) were selected randomly for inclusion into the study; making a total of ten gandas. Jimma zone is situated at approximately 7.6599° N latitude and 36.8327° E longitude, has a tropical wet and dry or savanna climate. The district’s yearly temperature is 15.28°C (59.5°F). Jimma typically receives about 91.72 millimeters (3.61 inches) of precipitation and has 194.96 rainy days (53.41% of the time) annually [52].

### Study population, sample size and technique

Our study population for survey was all selected spouses of heads of household who lived in the district for at least six month, whereas for the qualitative section diversified populations were selected purposefully. This study was part of a wider baseline investigation that sought to assess the effectiveness, feasibility and acceptability of co-delivery of two MDA for Onchocerciasis and STH. The sample size was determined using single proportion formula using 75% (effective campaign treatment coverage of STH) and considering design effect of 1.5, margin of error 4% and 10% non-response rate which gave 743.

The survey utilized a multistage sampling approach. Initially, five districts within the Jimma Zone were selected for the study. The selection of these districts was based on input from local health experts and consideration of the targeted endemicity of NTDs in the region. The sample was then proportionally distributed to selected districts. Then two ganda, which are the lowest administrative units in Oromia, Ethiopia were randomly selected from each of the selected districts. Similarly, the sample sizes were distributed proportionally to each district’s randomly selected ganda. Finally, a simple random sampling approach was used to select participants at the household level. This was done using census data which was conducted for the sake of sampling frame and aim of the main project. Further details have also been described in our previous study [53].

For qualitative part, a diverse population, including community members, school age children, frontline health workers, different experts and leaders at primary health care unit (PHCU) and district level were included purposefully to capture comprehensive information. The sample size was estimated based on the ultimate objective of the qualitative evidence required and the saturation of ideas was taken into consideration to stop further sampling. Recruitment of the participants was conducted considering different factors such as setting, gender, experiences and position. Accordingly seven key informant interviews (KII), six focus group discussion (FGD) and seven expert group discussions (EGD) were conducted. Besides, one group discussions were conducted with rural health extension workers. The participants of FGD were school boys, school girls, male adult community member, and female adult community member, whereas the participants of KII were ganda leaders, health extension workers and community drug distributors. Finally the participants of EGD were experts at PHCU and district levels including NTD experts and other health professionals working related to NTD, PHCU directors, school directors and health management information system focal.

### Data collection tool and procedure

A structured interviewer-guided administered questionnaire which was developed from relevant literatures and the qualitative study findings. It consisted of different parts including socio-demographic characteristics, knowledge, risk perception and preventive practices related to STH. Besides, water status, sanitation facility and hygiene practices were included.

For qualitative part, a semi-structured guide was developed by reviewing related piece of literatures considering the research objectives. It was developed by covering topics on STH its sign and symptoms, mode of transmission, consequences, preventive measures and WASH. It was collected by trained master-level experienced professionals (public health or social sciences) and good experience in qualitative research and who were fluent in the local language and were recorded using a digital voice recorder besides note-taking.

### Measurements and scoring procedures

#### Knowledge related to STH

Multi-dimensional knowledge about STH (consequences, symptoms, mode of transmissions and preventive measures) were measured using pertinent yes/no items. Accordingly, seven items for symptoms, eight items about the mode of transmission, five items about the consequences, and ten items about preventative measures were used. For each item, the correct response was given a score of 1 and incorrect responses assigned score of 0. The score was then summed up to produce separate indices for each knowledge dimension, namely knowledge of symptoms, mode of transmissions, consequences and protective measures. The overall (comprehensive) knowledge score was computed by summing up all the aspects of knowledge which consisted of thirty items, giving score values ranging from zero to thirty.

#### Perceived risk towards STH

It was measured using three item questions with three point response (agree, disagree, don’t know) format. For computing score, each response with agree were recoded as yes and given a score of 1 and otherwise recoded as no and given zero point. The score was then summed up to produce risk perception score which consisted of three items, giving score values ranging from zero to three.

#### STH preventive practice

It was measured using nine yes/no items (i.e. keeping food hygiene, keeping personal hygiene, taking medicine, washing hands before eating/preparing food, washing/cleaning fruit and vegetables before eating, boiling drinking water, adding aquatab in drinking water, proper utilization of latrine and washing hand after soil contact). For each item, the correct response was given a score of 1 and incorrect responses assigned score of 0. Then the score was summed up to produce preventive practice score giving a score of 0 to nine.

For standardizations and comparisons purpose, all scales (sub-knowledge score, overall knowledge score, risk perception score and preventive practice score) were rescaled to zero to ten values using Y = (X-X*min*)n/X*range* where Y is the rescaled variable, X is the original variable, X*min* is the minimum observed value on the original variable and X*range* is the difference between the maximum score and the minimum score on the original variable and n is the upper limit of the rescaled variable.

The mean value was used to divide the overall knowledge, its dimensions, and preventive practice into two categories: adequate if above the mean score and inadequate otherwise. The median value was used to divide the risk perception score in to high risk perception (above median value) and low risk perception (median and below median value).

### Frequency of hand washing practice at critical times

was measured by 8 likert scale questions (never, sometimes, often, and always) with a minimum score of 8 and a maximum score of 32. Then, the median score was used to categorize as adequate frequency of hand washing practice if the score is above median value and otherwise inadequate practice.

### Knowledge of hand washing at critical times

was measured using fifteen yes/no items. For each item, the correct response was given a score of 1 and incorrect responses assigned score of 0 and considered adequate if the scores were above the median, otherwise inadequate.

### Knowledge of proper technique of hand washing

In our study, participants who washed their hands with water and soap/ash at critical times were considered to have adequate knowledge of it.

### Household water treatment practice

was assessed by five yes/no items and coded as 1 if household practiced at least one of water treatment methods (adding medicine, boiling and cooling, filtering using clothes, using water filter and adding lemon) and 0 if not.

### Sanitation status

It was defined as ’improved sanitation’ if the participant’s household had access to one of the following: a pit latrine with a slab or platform, flush or pour-flush toilet, or a ventilated pit-latrine, which is not shared with other households and if not it was categorized under ‘unimproved sanitation’ [54].

### Latrine quality

was assessed using five observation check list (cleanness, having protected entry, having superstructure, having hand washing facility and presence of soap) and the summary score was computed (range 0 to 5) to quantify latrine quality. The study participants who had scored three out of five and above of score were categorized moderate quality and low quality otherwise [55].

### Data processing and analysis

The collected data were checked for completeness and cleaned, entered in to Epi data manager version 4.0.2 and then exported to SPSS version 21 for analysis. Following the standardization of the scores, descriptive statistics such as proportion, median and mean were computed to describe the findings. Multiple linear regressions were used to assess the association of the independent variables with overall knowledge and preventive practice adjusted scores. The assumptions of linear regressions were checked and were fulfilled.

Variables with a p-value less than 0.25 during the simple linear regression were selected for multivariable linear regression. Logistic regression was used to determine association between independent variables and frequency of hand washing practice at critical times. For the declaration of statistical significance, a 95% confidence interval and a level of significance of pvalue less than 0.05 were used. Kruskal-Wallis and Mann-Whitney tests were done to assess differences in median risk perception score between socio-demographic factors and statistical significance at pvalue <0.05 was used.

For qualitative part of the study, the audio data were transcribed verbatim and translated to English language. The coding and further analyses were assisted by Atlas.ti 7.1.4. The transcripts were read and re-read by the investigators who then allotted codes to all transcripts. An inductive approach of thematic analysis was used and the data were coded, categorized and thematized. Relevant and representative direct quotes were used to illustrate the interpretations, to explain and clarify the quantitative results.

### Data quality control

Experienced data collectors who were fluent in the local language collected the data. Public health experts reviewed the tool to ensure its relevance and content validity. Prior to the actual data collection, the tool was pretested to ensure its clarity and appropriateness in the local context, and necessary revisions were made. Data collectors received training and were supervised during the data collection. Various techniques were used to ensure the dependability, credibility, transferability, and confirmability of the qualitative findings. The data was triangulated by collecting through interviews and discussions from diversified study participants. Moreover, during and at the end of each interview and discussion, key points were summarized by the facilitators and the participants were requested to provide feedback and confirm whether the summaries reflected their ideas.

### Mixing of the quantitative and qualitative data

We first collected and analyzed qualitative data to initially explore a phenomenon which followed by quantitative data collection for quantification. The data were integrated through being connected between the qualitative data analysis and then quantitative data collection and finally integrated (S1 Material).

## Results

### Socio-demographic characteristics of the respondents

In this study, 732 households participated giving response rate of 98.5%. Five hundred and ninety five (81.3%) of the survey respondents were females. The respondents were predominately farmers (94.1%) and of Oromo ethnicity (88.5%) (Table 1).

**Table 1:** Socio-demographic characteristics of participants in Jimma, Oct_2021.

Variables	Category	Frequency (n = yes)	Percent
Sex	Male	137	18.7
	Female	595	81.3
Age (year)	15–24	106	14.5
	25–34	194	26.5
	35–44	216	29.5
	>=45	216	29.5
Role in house holds	House wife	576	78.7
	Husband	124	16.9
	Member	32	4.4
Marital status	Married	646	88.3
	Widowed	43	5.9
	Others ^a^	43	5.9
Educational status	No formal education	409	55.9
	Primary education	277	37.8
	Secondary education	46	6.3
Religion	Muslim	638	87.2
	Orthodox	66	9.0
	Others ^b^	28	3.8
Ethnicity	Oromo	648	88.5
	Amhara	31	4.2
	Others ^c^	53	7.2
Occupation	Farmer	689	94.1
	Others ^d^	43	5.8
District	Omo Nada	187	25.5
	Omo Beyam	131	17.9
	Kersa	137	18.7
	Gomma	162	22.1
	Manna	115	15.7

^*a*^(28single, 6separated, 9divorced);

^*b*^(26protestant, 2other);

^*c*^(25Hadiya, 9Dawuro,4Yem, 8Kafa,7other);

^*d*^(8merchant or private business,13 daily laborer, 1NGO worker,3pastoralist)

### Perceived susceptibility to STH

**Fig 1:**
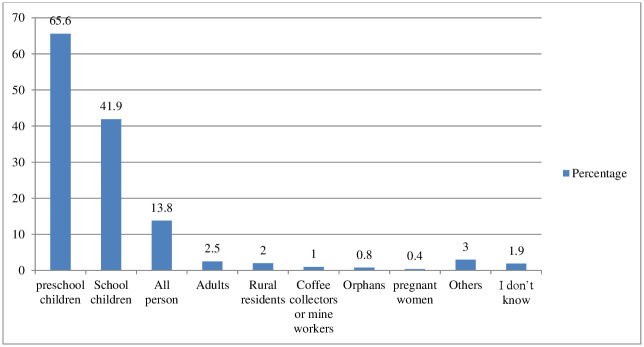
Percentage of knowledge on perceived susceptivility to STH in Jimma, 2021. **Others** [3(Poor hygiene individuals, malnourished individuals), 2(Stunted individuals, older individuals, less than 10 years, less than 1 year), 1(> 40 years, >6 months children, 2–10 years children, less than 2 years, less than 4 years, less than 30 years, mothers, poor segment of the community, urban residents)].

Almost all of the respondents (99.6%) reported that they had ever heard (a disease STH). Pre-school (under-five age) (65.6%) and school age (5–14 years old) (41.9%) were perceived to be most at risk of STHI (Fig 1).

Qualitative result revealed STH is locally called “ascariasis” or “germ” or “intestinal parasites.

*“…. Community do not directly call helminthes, they call it “Germ” (KII, HEW, district).*

The community recognizes this issue as their own, emphasizing that children are particularly vulnerable to the infection.

*“…….yes, it is a problem; since it affects any person, children to adults (FGD, P4, district).*

### Knowledge on sign and symptoms of STH

The study indicated that nearly half, 49.5%: (95%CI: 45.6, 52.9) of the participants had adequate knowledge on symptoms of STH. The commonest symptoms reported were intensive abdominal pain or cramp (84.0%), nausea and vomiting (45.2%), loss of appetite (43.3%) and diarrhea (40.4%) (Table 2).

**Table 2:** Knowledge on sign and symptoms of STH in Jimma, Oct_2021.

Characteristics	Frequency (n = yes)	Percentage
Abdominal pain or cramp	615	84.0
Nausea and vomiting	331	45.2
Loss of appetite	317	43.3
Diarrhea	296	40.4
Abdominal distension	244	33.3
Presence of parasite in the stool	197	26.9
Fatigue	36	4.9
Irritability	30	4.1
Itching anal area	19	2.6
Others*	85	11.6
I don’t know	8	1.1

*14(increase salivation),9(body swelling), 5(abdominal shouting), 4(dysentery, skin rash), 3(cause hunger, wasting, headache), 2(change color, no symptoms, fever, may expel per mouth, frequent strain to defecate, face swelling),1(make taste of mouth bitter, blindness, can be change to other disease. e.g cancer, cause other disease, change physical appearance, bedridden, damage intestine, depression, economic crisis, ‘garaa ruurressa’, ‘gar-malee luqqisa’, infection around anal area, death, abdominal burn, change face, fainting, cough, constipation, peptic ulcer disease, cools body, shorten people’s life, if severe it causes rash “cittoo”, thirsty, skin itching, reddish of hair, make the body greyish, odour change, cut the body, phobia of sound and sunlight, pain of kidney).

Qualitative finding was also supportive of quantitative results. The majority of the participants described that gastrointestinal symptoms such as abdominal pain, abdominal distention, diarrhea, vomiting and loss of appetite are the common symptoms of STH. “…*intestinal worms cause loss of appetite*, *vomiting*, *and abdominal distention*.*…” (FGD*, *P7*, *district)*.

### Knowledge on mode of transmissions of STH

The study indicated that half of the participants 50.8%: (95%CI:47.3,54.3) had adequate knowledge on mode of transmission and they largely attributed its causation to drinking contaminated water (73.4%), eating contaminated food (72.3%), poor personal hygiene (41.8%) and poor environmental sanitation (39.2%) (Table 3).

**Table 3:** Knowledge on mode of transmissions of STH in Jimma, Oct_ 2021.

Characteristics	Frequency (n = yes)	Percentage
Drinking contaminated water	537	73.4
Eating contaminated food	529	72.3
Poor personal hygiene	306	41.8
Poor environmental sanitation	287	39.2
Eating raw or unwashed fruits and vegetables	126	17.2
Preparing or eating food without washing hands	68	9.3
Touching soil	62	8.5
Eating too much cabbage	51	7
Lack of using toilet	42	5.7
Eating sweat food	40	5.5
Eating raw meat	33	4.5
Touching feces	9	1.2
Walking barefoot	8	1.1
Others*	36	4.9
I don’t know	26	3.6

*7(eating corn), 6(malnutrition), 4(eating enset),3(raw milk, lack of balanced diet),2(hunger, eating cool food), 1(add lemon to fruit and vegetables, allergy, drinking well and stored water, eating cabbage without oil, eating food that last a long time, eating food without salt, extra feeding for<6 months children, evil eye, find by nature in human abdomen)

Findings from qualitative participants indicated that drinking contaminated water is the cause or risk factor for STH. The study participants also repeatedly mentioned that poor food handling such as eating contaminated food, raw fruits or vegetables with poor hygiene, leftover food, spoiled food and food poisoning are the cause for STH or germs.*“…*…*Unsafe water is the cause of intestinal worms*. *Again*, *using contaminated food as well*. *Further*, *if a person eats food before washing hands a person can be caught an intestinal worm” (FGD*, *P1*, *district)*.

Majority of the participants stated that poor environmental sanitation and hygiene such as not using toilet or open defecation, not washing hand with soap and eating without hand washing, and poor waste management are the causes for intestinal worms. *“…It is from sanitation and hygiene*, *defecating on the field*, *not using toilets*, *related to environmental hygiene*, *not hand washing with soap …” (FGD*, *P8*, *district)*.

There were different views among some of the study participants regarding the mode of transmission of STH. They explained that contacts such by touching contaminants, from person to person and by using toilet contaminated by sick individuals. However, some of the study participants argued that STH can’t transmit from person to person.

“…. *This intestinal worm affects children then transmitted to older children……”(FGD*, *P4*, *district)*.

*“……I don’t think intestinal worms can transmit from one person to the other person” (FGD, P1, district).*

Some of the participants of FGDs also mentioned that bite by mosquito, eating sweetened food and flies transmits intestinal worms.

*“Mosquito may transmit the disease by biting healthy individuals after exposure to a sick person; through blood contamination” (FGD, P5, district)*

*“…a person affected by this disease may contaminate the toilet when defecating at the opening of the latrine. Then, flies may contact, so that it carries the disease and transmit it to others” (FGD, P4, district)*

One participant also perceived that blood donation may transmit intestinal worms.

*“….. If a person affected by this disease donates blood, he/she may transmit the disease to the other person” (FGD, P5, district)*

### Knowledge on consequences of STH

Half of the study communities; 50%:(95%CI:46.2, 54.1) had adequate knowledge on the consequences of STH with the main known harmful effects as stunting (43.6%) followed by death (37.4%) (Table 4).

**Table 4:** Knowledge on consequences of STH in Jimma, Oct_2021.

Characteristics	Frequency (n = yes)	Percentage
Stunting	319	43.6
Death	275	37.5
Damage internal organs	122	16.6
Anemia	81	11.1
Wasting	14	1.9
Don’t have negative consequences	8	1.1
Mental retardation	5	0.7
Poor school performance	7	1.0
Others*	61	8.3
Don’t know	24	3.3

*14(loss of appetite), 8(abdominal distension, body swelling), 3(face swelling, expel per mouth, bloody stool), 2(change color, Economic crisis), 1(‘garaa ruurresa, ‘garmalee luqqisa’, headache, can be change to other disease. e.g cancer, cause other disease, change physical appearance, blindness, made taste of mouth bitter, bedridden, depression, infection around anal area, cools body, shorten people’s life, vomiting, weakness, sparsing hair, abdominal cramp)

Qualitative finding also revealed that death was the consequence of STH mentioned by the study participants. One participant of FGD mentioned that STH could lead to invisible complications such as internal organ damage.

*“Although the community has no awareness, the intestinal worm is… fatal among children. ….there might be peoples who die due to the problem” (FGD, P5, district).*

### Knowledge on preventive methods of STH

Nearly half 47.5%:(95%CI:43.7, 51.4) of the communities had adequate knowledge on the preventive measures of STH and frequently mentioned keeping hygiene of food (63.9%) and keeping personal hygiene (50.7%) as preventive measures (Table 5).

**Table 5:** Knowledge on preventive methods of STH in Jimma, Oct_2021.

Characteristics	Frequency (n = yes)	Percentage
Keeping hygiene of food	468	63.9
Keeping personal hygiene	371	50.7
Taking medicine	284	38.8
Washing hands before eating food	245	33.5
Washing hands before preparing food	225	30.7
Boiling drinking water	167	22.8
Avoiding unwashed fruits/vegetable eating	95	13.0
Adding aquatab in drinking water	64	8.7
Washing hands after toilet	55	7.5
Proper utilization of toilet	39	5.3
Washing hands after touching soil	26	3.6
Avoiding eating raw meat	25	3.4
Avoiding eating sweet food	24	3.3
Proper disposal of wastes	7	1.0
Wearing shoes	5	0.7
Others*	68	9.3
Don’t know	22	3.0

*16(clean drinking water),10(eating balanced food), 7(keep material clean), 3(traditional medicine), 2(adding lemon to food), 2(adding lemon to water), 2(eating hot foods), 1(boiling milk, adding salt to food in good ways, avoidance of eating cool food, avoiding starvation, covering drinking water adequately, breast feeding(6mos), feeding butter at birth, covering the hole of toilet, destroying stagnant water, toilet hygiene, well cooking food, drinking tella and alcohol, drinking well water, eating cabbage, eating chills, eating food containing salt and chills, eating fresh food, eating spicy food, exclusive breast feeding, isolation, environmental sanitation, not eating too much cabbage, vaccination, wash hand with soap/ash, using clean water for preparing food, taking oncho drug)

The participants of FGD and KII explained that keeping personal (especially children’s) and environmental sanitations and hygiene such as appropriate toilet utilization and washing hands with soap or ash and water, and improving solid and liquid waste management helps to prevent STH.

*“….It is right that if we keep our hygiene and properly use our toilet, we can prevent ourselves and our children from ascariasis [intestinal worms]. Hygiene should be from home to toilet; if we fail to keep our hygiene, our children’s hygiene, and sanitation of our environment including drinking water, we can be infected by ascariasis” (FGD, P1, district).*

The study participants also suggested that improving food handling, avoiding sweet foods prevents intestinal worms. They also stated that protection and treatment of drinking water by boiling, adding wuha Agar and adding lemon into drinking water enable the community to prevent worms.

“…. *if possible*, *adding water treatment [wuha agar]*, *if not adding lemon into drinking water helps to clean water and prevent intestinal worms……*. *Thus*, *we able to prevent the worm who came from different fluids” (FGD*, *P2*, *district)*

Findings obtained from participants of FGDs showed that taking medications is the solution for prevention of intestinal worms.

“*I think the drug is a solution …*, *to all people living in rural areas very far from a health facility*. *Therefore*, *distributing the drug to all community members is a solution (FGD*, *P7*, *district)*.

### Perceived risk and severity perception towards STH

**Fig 2:**
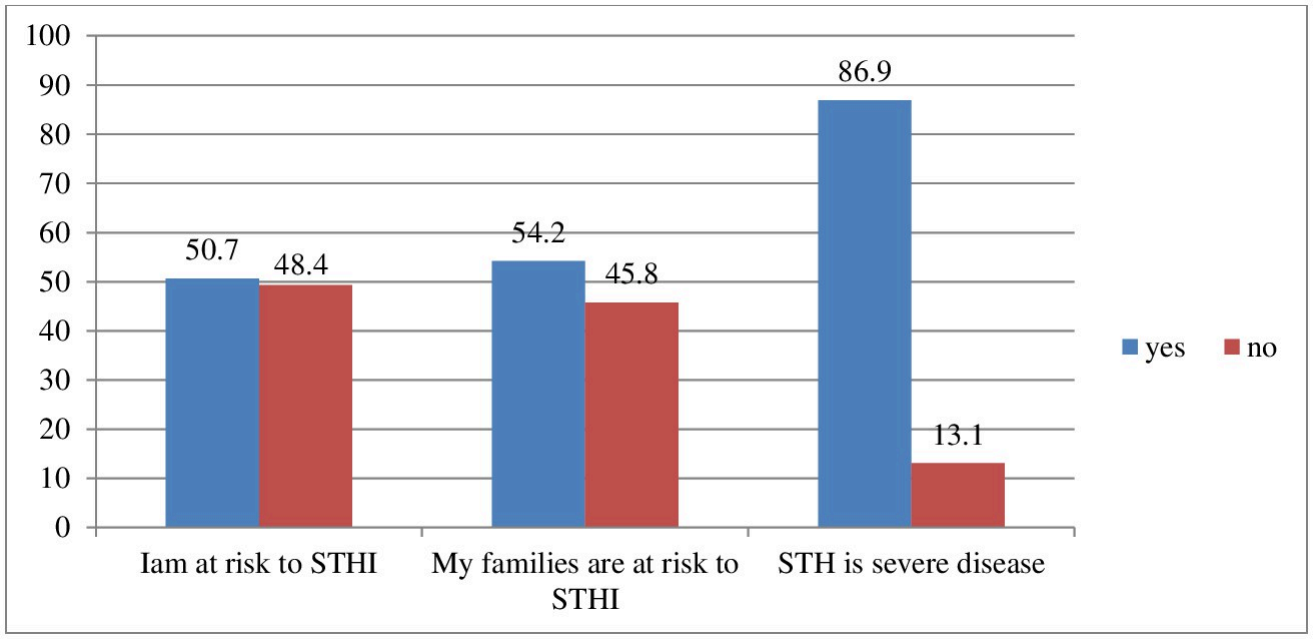
Percentage of risk perceptions of the respondents towards STH in Jimma, Octobe_2021.

Six hundred thirty six (86.9%) of the respondents perceived that STH is a severe disease. Half of the participants (50.7%) had perceived risk of getting infected by the STHI (Fig 2).

### Median risk perception score comparison

The average risk perception score was varied significantly among the groups of educational status (p = 0.002), study district (p = ≤0.001) and age category (p = 0.015) according to the Kruskal-Wallis test (Table 6).

**Table 6:** Kruskal-Wallis Test and Mann-Whitney U Test of risk perception average score variations by Sociodemographic characteristics, Oct_2021.

Parameters	Frequency n(%)	Chi-square value	P-value
**Educational level**			
No formal education	409(55.8)	12.83	0.002**
Primary education	277(37.8)		
Secondary education	46(6.3)		
**Study district**			
Omo Nada	187(25.5)	26.51	≤0.001**
Gomma	162(22.1)		
Omo Beyam	131(17.9)		
Kersa	137(18.7)		
Manna	115(15.7)		
**Age category (year)**			
15–24	106(14.5)	10.46	0.015**
25–34	194(26.5)		
35–44	216(29.5)		
> = 45	216(29.5)		
**Ethnicity**			
Oromo	648(88.5)	2.50	0.285*
Amhara	31(4.2)		
Others	53(7.2)		
**Religion**			
Muslim	638(87.2)	3.79	0.15*
Orthodox	66(9)		
Others	28(3.8)		
Mann-Whitney U Test			
**Sex**		Z value	
Male	137(18.7)	-1.259	0.208*
Female	595(81.3)		

**Value statistically significant,

* Value statistically not significant

### Preventive practices of STH

The study indicated 44.4% of the study participants had adequate practice on preventive measures of STH and keeping food hygiene 550(75.1%), keeping personal hygiene 441(60.2%) and washing hands before eating/preparing food 234(32%) were practiced most frequently (Table 7).

**Table 7:** Preventive practices of the respondents on STH in Jimma, Oct_2021.

Characteristics	Frequency (n = yes)	Percentage
Keeping food hygiene	550	75.1
Keeping personal hygiene	441	60.2
Washing hands before eating/preparing food	234	32.0
Boiling drinking water	158	21.6
Washing/cleaning fruit and vegetables before eating	139	19.0
Washing hands after toilet	80	10.9
Adding wuha agar to drinking water	57	7.8
Proper utilization of latrine	38	5.2
Taking medication	39	5.3
Washing hand after soil contact	34	4.6
Avoiding eating raw meat	30	4.1
Avoiding eating sweet foods	29	4.0
Waste disposal utilization	15	2.0
We are doing nothing	59	8.1
Others*	72	9.8
I don’t know	6	0.8

*29(maintaining water quality), 7(cleaning materials), 3(eating balanced diet), 4(adding lemon to water),2(eat chills, drinking pipe water, un avoidable, eat cabbage with oil, eating food after hot it, avoid drinking stored water, eat fatty food), 1(eating balanced diet, covering water storage, drinking lemon juice, Eating spicy food, environmental sanitation, filtering water using clothes, keeping toilet hygiene, drinking protected spring water, educating our children, no eating cabbage, prevented by ‘rabbi’, mixing and boiling of sugar and lemon, traditional medicine, using onion, eating hot food)

### Overall knowledge, its dimension and preventive practice scores

**Fig 3:**
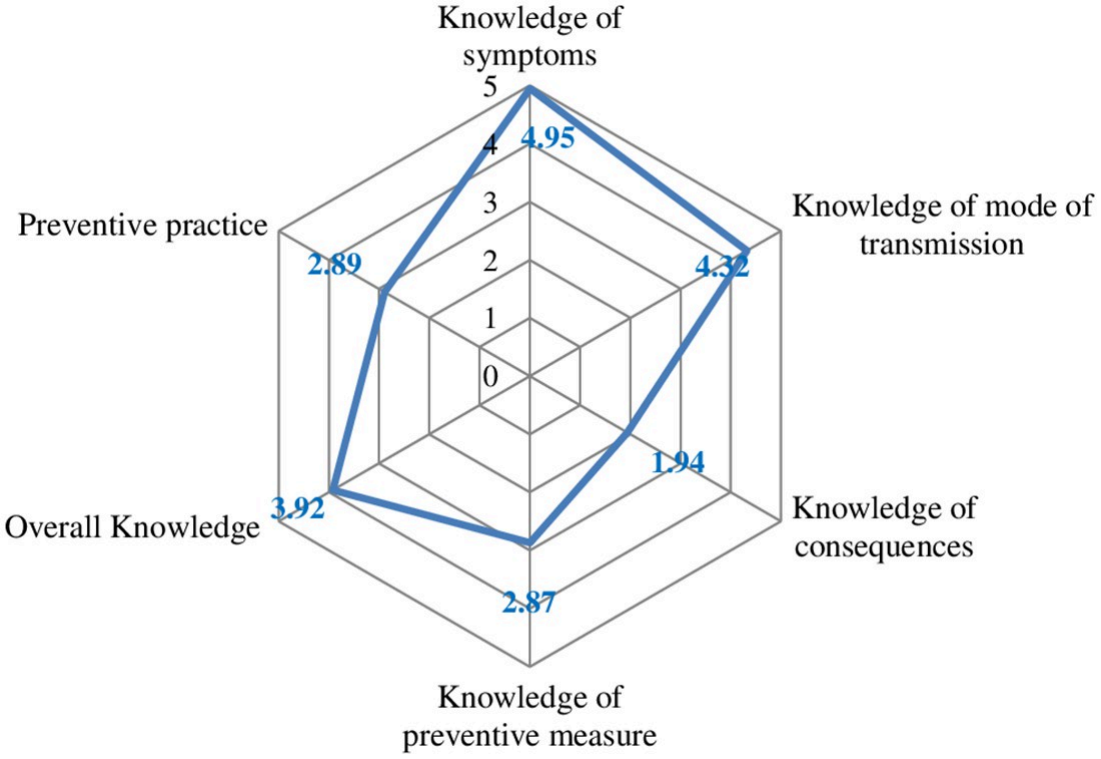
Mean score on knowledge and preventive behaiviors towards STH, Jimma, Oct_2021.

After the score was computed and rescaled to (0–10 score), the mean value used to split each score dimension and the results are plotted in radar chart (Fig 3). The highest mean score was the knowledge of symptoms and the lowest was on the knowledge of consequences.

### Prevalence of overall knowledge

**Fig 4:**
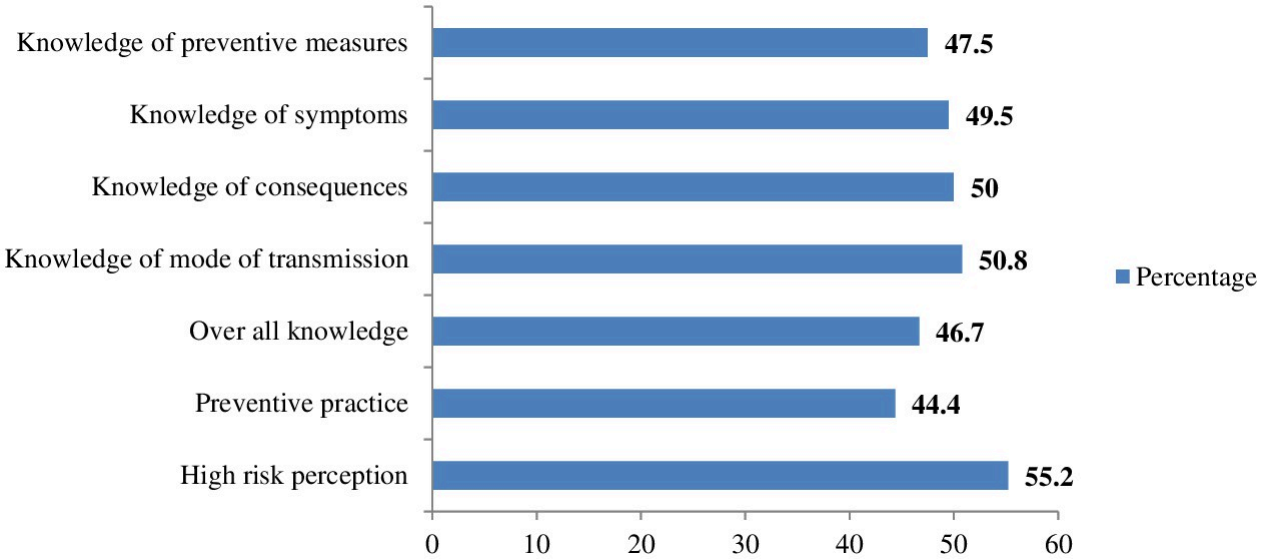
Percentage of overall knowledge, its dimensions, high risk perception and preventive practices towards STH.

The prevalence of adequate overall knowledge, high risk perception and adequate preventive practices towards STH were 46.7%: (95%CI: 43.2, 50.4), 55.2%: (95%CI: 51.2, 59) and 44.4%: (95%CI: 40.8, 48.2) respectively (Fig 4).

### Factors associated with comprehensive knowledge towards STH

Study district, educational status and latrine status were statistically significant with comprehensive knowledge of STH. Respondents who achieved secondary education had an average increase of 0.71 in overall knowledge (β = 0.71, (95%CI: 0.22, 1.21) compared with those who had no formal education after controlling all other predictors. Likewise, respondents who had improved latrine were about 0.38 average increase in comprehensive knowledge (β = 0.38,(95%CI:0.14, 0.61) when compared with their counterparts after controlling all other predictors. Finally, participants live in Omo Nada and Manna districts were an average increase of 0.65 and 0.42 respectively in their STH comprehensive knowledge compared with O/Beyam district (p-value <0.05) (Table 8).

**Table 8:** Factors associated with comprehensive knowledge towards STH in Jimma, Oct_2021.

Variables	Category	N (%)	VIF	P-value	Unstandardized B coefficient	95% CI for B
	Constant			≤0.001	3.30	(2.9, 3.6)
District	Omo Nada	187(25.5)	1.8	<0.001*	0.65	(0.32,0.98)
	Gomma	162(22.1)	1.9	0.218	0.22	**
	Omo Beyam	131(17.9)	1	1	1	1
	Kersa	137(18.7)	1.7	0.074	0.32	**
	Manna	115(15.7)	1.8	0.038*	0.42	(0.02, 0.81)
Marital status	Married	646(83.3)	1	1	1	1
	Widowed	43(5.9)	1.1	0.796	-0.062	**
	Others	43(5.9)	1.2	0.775	-0.071	**
Educational status	No formal education	409(55.9)	1	1	1	1
	Primary education	277(37.8)	1.3	0.620	-0.063	**
	Secondary education	46(6.3)	1.3	0.005*	0.71	(0.22, 1.21)
Age (year)	15–24	106(14.5)	1	1	1	1
	25–34	194(26.5)	2.3	0.086	0.320	**
	35–44	216(29.5)	2.6	0.225	0.233	**
	> = 45	216(29.5)	2.6	0.610	-0.098	**
Proper solid waste disposal	No	673(91.9)	1	1	1	1
	Yes	59(8.1)	1.1	0.733	0.071	**
Latrine status	Unimproved	471(64.3)	1	1	1	1
	Improved	261(35.7)	1.1	0.002*	0.38	(0.14, 0.61)

*value statistically significant;

**: value not statistically significant; 1: reference; VIF: Variance inflation factor

### Factors associated with preventive practice towards STH

Study district, having adequate comprehensive knowledge of STH and adequate knowledge of hand washing at critical times were statistically significant with preventive practice of STH. Participants live in Omo Nada, Kersa and Manna districts were an average increase of 0.1, 0.09 and 0.12 respectively in their STH preventive practice compared with O/Beyam district. Moreover, participants who had adequate hand washing knowledge at critical times had an average increase of 0.29 unit in their STH preventive practice compared to those with inadequate knowledge of it (95%CI: (0.76, 1.21). Finally, participants who had adequate knowledge on comprehensive knowledge of STH had an average increase of 0.32 unit in their STH preventive practice compared to those with inadequate knowledge (95%CI: (0.85, 1.29) (Table 9).

**Table 9:** Factors associated with preventive practice towards STH in Jimma zone, Oct_2021.

Variables	Category	N (%)	VIF	P-value	Unstandardized B coefficient	95% CI for B
Constant				≤0.001	1.738	(1.285, 2.190)
Sex	Female	595(81.3)	1	1	1	1
	Male	137(18.7)	1.1	0.756	0.001	**
Religion	Muslim	638(87.2)	1	1	1	1
	Orthodox	66(9.0)	2.4	0.519	0.031	**
	Others	28(3.8)	1.9	0.480	0.031	**
Ethnicity	Oromo	648(88.5)	1	1	1	1
	Amhara	31(4.2)	2.0	0.984	0.001	**
	Others	53(7.2)	2.3	0.195	-0.062	**
Study district	Omo Nada	187(25.5)	2.1	0.026*	0.10	(0.05, 0.72)
	Gomma	162(22.1)	2.1	0.318	0.045	**
	Omo Beyam	131(17.9)	1	1	1	1
	Kersa	137(18.7)	1.9	0.048*	0.09	(0.004, 0.72)
	Manna	115(15.7)	2	0.007*	0.12	(0.15, 0.94)
Age (year)				0.186	-0.046	**
Educational status	No formal education	409(55.9)	1	1	1	1
	Primary education	277(37.8)	1.3	0.117	0.056	**
	Secondary education	46(6.3)	1.2	0.171	0.048	**
Hand washing knowledge	Inadequate	421(57.5)	1	1	1	1
	Adequate	311(42.5)	1.2	≤0.001*	0.29	(0.76,1.21)
latrine quality	Low	592(80.9)	1	1	1	1
	Moderate	140(19.1)		0.057	0.068	**
Latrine status	Unimproved	471(64.3)	1	1	1	
	Improved	261(35.7)	1.4	0.886	-0.005	**
Proper solid waste disposal	No	673(91.9)	1	1	1	1
	Yes	59(8.1)	1.1	0.586	0.018	**
Overall knowledge of STH	Inadequate	390(53.3)	1	1	1	1
	Adequate	342(46.7)	1.2	≤0.001*	0.32	(0.85, 1.29)

*value statistically significant, 1: reference; VIF: variance inflation factor

### Water, Sanitation and Hygiene Practices

Just 20.8% of the participants used water treatment, despite the fact that only 14.8% of them had access to pipe water. Thirty five point seven percent and 29.8% cover and cleaned the water storing equipment respectively and boil the water (10.1%) to keep water clean for drinking whereas 46.2% of households were not doing anything to keep their drinking water hygienic (Table 10).

**Table 10:** Water treatment practices in Jimma, Oct_2021.

Characteristics	Number (n = yes)	Percentage
**Water treatment practices**		
Boiling and cooling	63	10.1
Adding medicine like bishan gari, wuha agar, aqua fresh	53	8.5
Filtering using clothes	28	4.5
Use water filter	18	2.9
Adding lemon to the drink water	16	2.6
**Other water cleaning practices**		
Covering the water storing equipment daily	223	35.7
Daily cleaning water storing equipment	186	29.8
Do nothing	288	46.2
Don’t know	11	1.8
Others*	6	0.08

*1(adding salt, storing water, cleaning the drinking water environment, not storing water for a longer period, cleaning drinking instruments, let it sit and filter)

The qualitative study participants explained that there was poor access to drinking water both for the communities and public institutions. The participants highlighted that communities lack clean drinking water and in some areas, they drink water from the river and are in great fear for their health. Findings also showed that majority of key public institutions such as health facilities and schools had no drinking water or any type of water at all. The community highly needs Wuha Agar for water treatment however they were usually facing shortage.

*“As you can see this community is living in the rural area where rivers are used as drinking waters. Community fears drinking contaminated water, they know it could be the source of worms…I have been teaching the community on the methods of water purification like filtering with clean cloth, boiling before drinking and using for other purposes, and adding the extract of lemon” (KII, HEW, district).*

Another participant stated, *“Lack of water is very common at school*, *where it is most needed…” (EGD*,*P2*, *district)*.

### Sanitation facility

**Fig 5:**
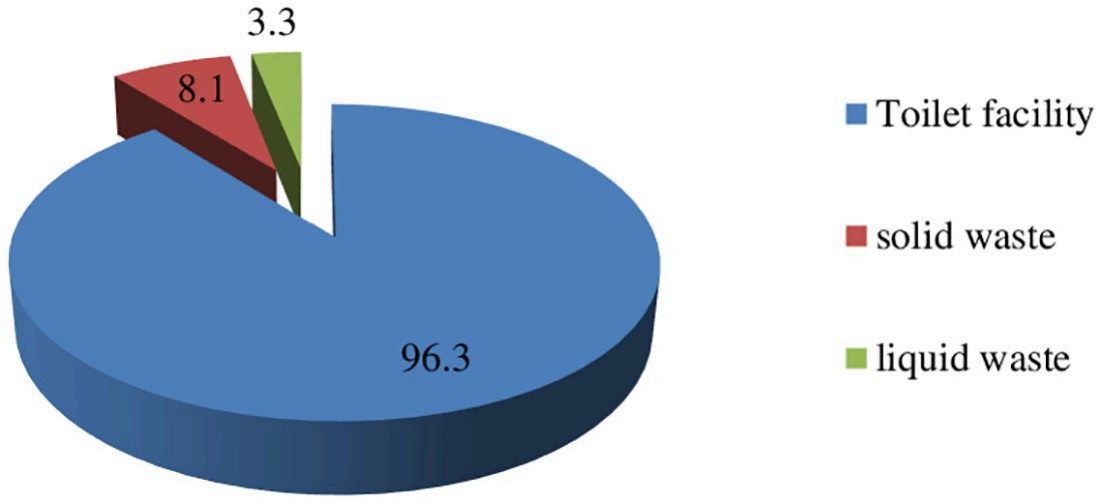
Percentage of toilet availability and proper waste disposal status Jimma, 2021.

The majority of participants (96.3%) had a toilet, while 8.1% had a solid waste disposal facility and only 3.3% had a liquid waste disposal facility (Fig 5).

### Type, quality, utilization and perceived benefits of latrine

The common type of latrine used by the study participants were toilet with only hole slab with no shelter 58.6% and pit latrine 35.2%. Besides, 94.7% households use private toilet (Table 11).

**Table 11:** Sanitation facility status of the study participants in Jimma, Oct_2021.

Type	Frequency (n = yes)	Percentage
Toilet with only hole slab but, no shelter	429	58.6
Pit-latrine with slab, but no water	258	35.2
Improved ventilated latrine	5	0.7
Flush or pour flush toilet	3	0.4
Not willing to observe	13	1.8
Overall improved sanitation	261	35.7
**Sanitation practices of under five children**		
To the toilet	280	61.1
On street	97	21.2
Burying	47	10.3
In the garden	18	3.9
Don’t have child	274	37.4
Others*	11	2.4

*(4) use cloth, (4) prepare well separately, (3) throw to canal or stream water

### Perceived benefit of toilet and its quality

The utilization of toilet was perceived by the households mainly to keep environmental and personal hygiene 63.4%, prevent communicable diseases 56.1 and prevent from flies 48.1% (Table 12). By observation of the toilet only 19.1% was in moderate quality.

**Table 12:** Perceived benefit and latrine quality status on observation in Jimma, Oct_2021.

Characteristics	Frequency (n = yes)	Percentage
**Perceived benefit**		
To keep environmental and personal hygiene	464	63.4
To prevent communicable disease	411	56.1
To prevent different flies	352	48.1
To protect families health	230	31.4
To keep privacy	103	14.1
To prevent water contamination	21	2.9
Don’t know toilet benefits	9	1.2
Others*	10	1.3
**Quality of latrine by observation**		
The toilet is clean	171	23.4
The toilet has protected entry	304	41.5
The toilet has superstructure	245	33.5
The toilet has hand washing facility	46	6.3
The toilet has soap	27	3.7

*(7)To remove bad smell, (1)for beauty, (2)prevent different germs and worm

Qualitative findings showed that even though the quality was not as per expectation, sanitation coverage or access and practices have improvement in the community.

*“At a community level the problem is on having a standard latrine; even though it is not standard; a majority has a latrine” (EGD, district NTD expert).*

The participants repeatedly explained that the majority of the households have simple pit type of latrine which was constructed of locally available materials such as grass, straw, insecticide treated net, or sometimes pit only without any covering materials. The construction of pit latrines was not assisted by professionals and as a result it was constructed at inappropriate sites and collapse after few periods of time. Findings showed that the simple pit latrine lacks cleanness and only few households had standardized latrine which contain slab, house, soap and water for hand washing.

*“…. The community constructs the latrine and uses it. At least they construct it with a mosquito net or other cover. But it is not clean, it could not prevent rain, some of them just cover with mosquito nets, and some other had no superstructure” (KII, HEW, district).*

Another participant also stated,

*“There are also some problems regarding the place where the toilet is built; some households built it in front of their door, and some others built it very far from their home” (KII, HEW, district).*

The participants of the study also mentioned that there is improvement of public latrine coverage constructed near main roads.

*“Latrine is found everywhere. There was a community latrine around main roads in some areas. Again, every household has a private latrine….” (FGD, P2, district).*

### Latrine utilization behavior

Regarding latrine utilization, findings obtained from FGDs and KIIs showed the improvement of utilization and in some areas open defecation is considered as taboo and they are moving towards open defecation free Gandas. However, latrine utilization behavior was quite far from the standards especially during summer time.

*“…Yes they are using, even nowadays defecating in the field is a social taboo in this kebele. However, there are still very few individuals who constructed toilet only to fulfil the criteria that set by health extension workers” (KII, HEW, district)*

Another participant also stated,

“When we see sanitation and hygiene, there is a good improvement than ever before… Previously when we move in a bush, we see defecation here and there” (FGD, P7, district)

### Perceived benefit and knowledge of proper way of hand washing at critical times

Most hand washing was practiced for the perceived benefit of keeping hygiene 83.1% and prevent diseases 69.4% (Table 13).

**Table 13:** Perceived benefit of hand washing among participants in Jimma, Oct_2021.

Characteristics	Frequency (n = yes)	Percentage
To keep hygiene	608	83.1
To prevent diseases	508	69.4
To prevent Covid-19	58	7.9
For personal comfort	43	5.9
**Knowledge of proper hand washing way at critical times**		
Washing with water and soap/ash	701	95.8
Hand washing with water only	15	2.0
I don’t know	10	1.4
Washing five times a day	1	0.1
Had proper knowledge	701	95.8

### Knowledge of hand washing at critical times

More than three quarter of study participants (83.2%) and 70.5% reported it is necessary to wash hands before eating and preparing meals respectively (Table 14). The overall prevalence of adequate knowledge of hand washing at critical times was 42.5%: (95%CI: 38.7, 45.9).

**Table 14:** Knowledge of hand washing at critical times in Jimma, Oct_2021.

Characteristics	Frequency (n = yes)	Percentage
Before eating food	609	83.2
Before preparing meal	516	70.5
After eating food	483	66.0
After toilet	435	59.4
After cleaning dung	284	38.8
During praying	237	32.4
After coming from outside	226	30.9
Before touching food	171	23.4
After cleaning child’s feces	124	16.9
Before feeding child	106	14.5
After touching soil	92	12.6
Before fetching water	25	3.4
After touching pets(dog, cats)	14	1.9
Before touching eye, nose and mouth	13	1.8
After touching eye, nose and mouth	6	0.8
Others*	13	1.7

*5afterwork,1after returning from market, after preparing food, before chewing khat, before and after work, before breastfeeding, When return from abroad, While drinking coffee, while sweating

### Frequency of hand washing practice at critical times

Regarding frequency of hand washing most households always wash their hands after toilet 88.5%, before preparing, touching and serving meal 82.4% and before eating food or feeding child 81% (Table 15). The overall prevalence of adequate practice of hand washing at critical times was 43.9%: (95%CI: 40, 47.5).

**Table 15:** Frequency of hand washing practice at critical times in Jimma, Oct_2021.

Items	Always N (%)	Often N (%)	Sometimes N (%)	Never N (%)
After toilet	648(88. 5)	56(7.7)	23(3.1)	5(0.7)
After cleaning child’s stool	386(52.7)	56(7.7)	15(2.0)	275(37.6)
Before preparing, touching and serving meal	603(82.4)	88(12.0)	17(2.3)	24(3.3)
Before eating food or feeding child	593(81.0)	87(11.9)	22(3.0)	30(4.1)
After touching pets(dog, cat)	141(19.3)	70(9.6)	92(12.6)	429(58.6)
After cleaning dung	419(57.2)	169(23.1)	120(16.4)	24(3.3)
After touching soil	443(60.5)	162(22.1)	103(14.1)	24(3.3)
Before touching eye, nose and mouth	55(7.5)	47(6.4)	188(25.7)	442(60.4)

The qualitative study participants repeatedly mentioned that there was improvement of personal hygiene as well as environmental sanitation in the community. They stated that health extension workers and teachers are giving health education for the communities and students on personal and environmental sanitation.

*“Honestly speaking, more than 80% of our community are on good level of sanitation and hygiene practices……” (KII, HEW, district).*

However, some of the study participants quoted that personal hygiene especially those of children is still poor. In addition, they mentioned that the environmental sanitation and hygiene is poor and some of the communities are living with cattle in a house.

*“….the problem is about the hygiene of children” (FGD, P6, district).*

### Factors associated with hand washing practice at critical times

Nine variables (age, educational level, religion, sex, ethnicity, knowledge of hand washing, latrine status, proper solid waste disposal and overall knowledge on STH) showed evidence of association with the frequency of hand washing practice at critical times at a p-value of <0.25. Of those variables, knowledge of hand washing, latrine status and age were statistically significant with outcome variable. Participants who had adequate knowledge on hand washing were 1.4 times more likely to engage in hand washing practices at critical times than participants without that knowledge (AOR = 1.4,(95%CI:1.0,2.0). Moreover, compared with older peoples (≥45years), people 15–24 and 25–34 years of age were 2.1 times and 1.9 times more likely to frequently wash their hand at critical times respectively compared with their counterparts [AOR = 2.1, (95%CI:1.2,3.5), AOR = 1.9, (95%CI: 1.3,3.0)]. Finally participants who had improved latrine were 1.6 times more likely to wash their hand at critical times AOR = 1.6, (95%CI: 1.1,2.2)]. (Table 16).

**Table 16:** Factors associated with hand washing practices at critical times, Oct_2021.

Variables	Category	hand washing practices		P- value	COR (95%CI)	P-value	AOR (95%CI)
		inadequate	adequate				
Age (years)	15–24	47	59	≤0.001	2.5(1.5,3.9)	0.006*	2.1(1.2, 3.5)
	25–34	91	103	≤0.001	2.1(1.4,3.2)	0.001*	1.9(1.3, 3.0)
	35–44	125	91	0.030	1.5(1.0,2.3)	0.053	**
	> = 45	149	67	1	1	1	1
Sex	male	85	52	0.246	0.8(0.5, 1.2)	0.386	**
	female	327	268	1	1	1	1
Educational status	no formal education	242	167	1	1	1	1
	primary education	151	126	0.252	1.2(0.9,1.6)	0.665	**
	secondary education	19	27	0.024	2.0(1.1,3.8)	0.185	**
Ethnicity	Oromo	357	291	1	1	1	1
	Amhara	23	8	0.042	0.4(0.2,0.9)	0.351	**
	Others	31	22	0.633	0.8(0.5, 1.5)	0.464	**
Religion	muslim	351	287	1	1	1	1
	orthodox	45	21	0.011	0.5(0.3, 0.8)	0.225	**
	Others	16	12	0.787	0.9(0.5, 1.9)	0.863	**
Proper solid waste disposal	no	383	290	1	1	1	1
	yes	28	31	0.163	1.5(0.9, 2.5)	0.642	**
Latrine status	Not improved	286	185	1	1	1	1
	improved	125	136	0.001	1.7(1.2, 2.3)	0.006*	1.6(1.1,2.2)
Over all knowledge of STH	inadequate	235	155	1	1	1	*
	adequate	177	165	0.1	1.3(0.9,1.7)	0.618	**
Knowledge of hand washing	Inadequate	259	162	1	1	1	1
	adequate	153	158	≤0.001	1.7(1.3, 2.3)	0.031*	1.4(1.0,2.0)

*value statistically significant;

** value not statistically significant; 1: reference

## Discussion

Ethiopia has set an ambitious national target of eliminating STH, but it is still one of the most important public health problem [13]–[17]. For an intervention to be implemented effectively and to encourage the adoption of control measures that are essential to support elimination prospects of STH a clear understanding of local knowledge, perceptions, and practices about it is essential [48], [49]. In this study we assessed community knowledge, risk perception, and use of preventive practices regarding STH using a mixed method study for in depth understanding in Jimma zone. The findings revealed that almost all of the respondents (99.6%) had heard of STH which was supported by previous study [56] and locally called “ascariasis” or “germ” or “intestinal parasites”. However, the results showed that the participants’ knowledge status on STH was generally insufficient. Less than half of the participants (46.7%) in this study had adequate comprehensive knowledge, which require culture and context specific awareness raising interventions [57].

According to current study, knowledge of typical sign and symptoms of STH was generally insufficient as only nearly half of the respondents (49.5%) were able to adequately recognize its classic symptoms which is in agreement with the South Africa study [58]. However, it is higher than study done in Malaysia (29.3%) [59]. The probable justification might be due to the geographic and contextual variations accounted for the differences in local recognitions of STH. Abdominal pain, nausea, vomiting and diarrhea were the common symptoms reported and earlier studies also reported similar finding [59], [60]. In order to use early health seeking and take preventive action, it is crucial that the public remain well-informed about the typical symptoms of STH as one of the main objectives of public health education is to assist people in recognition of the disease based on its symptoms. This is because it motivates people to seek treatment at an early stage, which aids in the prevention and control efforts.

In this study, we found that only half of the participants (50.8%) had sufficient knowledge on the mode of transmission of STH and this low level of knowledge of STH transmission could place them at a risk of infection. This result is higher than study done in Malaysia 28.8%[59] and 32.7% in Cameroon[56]. The possible reasons for discrepancy might be differences in sample size used, socio-demographic differences, control programmes and operational definition used. For instance study conducted in Cameroon was measured by a single item and used lower sample size compared to our study. Indeed, dinking contaminated water, eating contaminated food and poor hygiene were reported frequently as the mode of its transmission which was supported by previous literature [59]. Surprisingly, walking bare foot and touching faeces were reported by less than 2% of participants and only 5.6% reported lack of using toilet as the mode of transmission of STH. Furthermore, current study revealed there are knowledge gaps and misconceptions on mode of transmission such as by touching contaminants, from person to person, eating sweetened food, by using toilet contaminated by sick individuals, bite by mosquito and blood donation perceived as the mode of transmission which was also reported in other studies [57], [61]. This implies that creating STH education programs that are context-specific and tailored to these local knowledge gaps where poor sanitation and hygiene conditions are prevalent are essential.

The study results showed that only 14.8% of the participants had access to pipe water and one out of five (20.8%) reported treatment of water for drinking purpose which was lower than the study in Rwanda, where 35% of respondents consumed untreated water [62]. Furthermore, participants of the qualitative study reported there is much problem with respect to availability of water for both communities and public institutions. Regarding sanitation, even though 96% of respondents reported they had a latrine facility, the percentage of respondents having improved sanitation and moderate-quality latrines were only 35.7% and 19.1% respectively. This finding is lower than the study from peri-urban of Jimma town (56.1%)[16]. This variation can be explained by differences in the study setting, socio-demographic characteristics and sample size. Additionally, roughly one out of five (21%) of the study participants to handle under-five children’s stool they use street, and 4% use their gardens for this purpose. The finding generally underscores the need for community awareness-raising interventions to equip with the necessary knowledge and an input for other stakeholders like health extension workers.

Our finding revealed that 50% of the participants had sufficient knowledge on the consequences of STH. In fact, the majority of participants stated stunting (43.6%) and death (37.5%) as its consequences. Moreover, in this study perceived severity was also high (86.9%). However, knowledge on how to prevent STH was generally insufficient (47.5%) which is in agreement with the previous findings 47.2% in Cameroon [56] and 51.2% in Abaye Deneba area, Ethiopia [63]. Keeping hygiene of food, keeping personal hygiene, taking medications were mentioned most as preventive measures which is supported in previous studies [59], [60]. Washing hands after touching soil and wearing shoes were reported by less than 4% of participants as the preventive measures in this study which needs attention.

In this study, generally the magnitude of adequate comprehensive knowledge on STH was 46.7% which is in line with studies conducted in Sekota, Amhara region, Ethiopia 45.2% [64] and Bule Hora town, Oromia 48.4% [65]. This implies that approximately 50% of the study participants lacked comprehensive understanding of STH, which may compromise the efficacy of control efforts since the body of evidence demonstrates the critical role knowledge plays in controlling and prevention of infectious diseases [66]. These highlight the necessity of developing health education programs and carrying out communication interventions for social and behavioral changes. This finding is higher than study done in Thailand 29.53% [67], whereas it is lower than studies conducted in Bete towns, North Shoa 60.3%, Ethiopia [68] and Indonesia 56.1%[69]. The variation might be due to the difference in operational definition used, study period, sample size, and socio-demographic characteristics. For example, study conducted in Indonesia was conducted on pregnant women who might have better exposure to health information including STH during their routine ante natal care follow up and classified their knowledge status into three categories whereas, study in Thailand used scores greater than 80 percent of the knowledge items to have good knowledge of STH.

Participants who completed secondary education were more knowledgeable about STH compared to those with no formal education. This is in line with other studies [70], [71]. This could be due to the fact that, educated individuals are likely to be more exposed to health information about STH. Furthermore, having improved latrine is associated with comprehensive knowledge of STH suggesting improving comprehensive understanding about STH could potentially contribute to construction and use of improved sanitation facility for STH prevention.

In this study, the magnitude of adequate preventive practices was 44.4% which is supported by other evidence in Bule Hora, Southern Ethiopia (46.9%) [65] and comparable with the magnitude of comprehensive knowledge in this study. It implies that most community members did not know and adhere to common preventive practices, which could be the result of widespread ignorance and a lack of appropriate information through well-coordinated health awareness programs in the area. In line with earlier studies our study showed comprehensive knowledge of STH is positively associated with STH preventive practice [67], [69]. This illustrates how knowledge is a crucial element of successful preventive strategies since it is a prerequisite for the behavior change to happen, and increased awareness fosters better practice. The notion that the public can make "informed decisions" about health behaviors by leveraging their knowledge is sound, even though it is difficult to determine how much knowledge is sufficient to achieve desired changes in health outcomes.

On the other hand, our finding is higher than study conducted in Thailand 33.51%) [67]. However, it was lower than study conducted in Sekota town, Amhara region Ethiopia 51.1% [64] and 54% in India [72]. The sample size difference, demographic characteristics, operational definition utilized, and study setting could be the contributing factors for the discrepancy. For instance, in contrast to our study, which used the median score, a study carried out in Thailand used scores greater than 80% of the practice items were considered to have good practices. In our study, the majority of participants mentioned keeping food hygiene, keeping personal hygiene, washing hands before eating or preparing food were used as preventive practices which is corroborated by earlier research [59].

The study’s findings suggest a significant disparity in both the comprehensive knowledge of STH and preventive practices across different districts. Respondents from the Omo Nada and Manna districts possess notably higher levels of knowledge about STH, with average increases of 0.65 and 0.42, respectively, compared to their counterparts in the O/Beyam district. This indicates that educational or awareness initiatives in these districts may have been more effective or that there may be other underlying factors contributing to their enhanced understanding.

Similarly, the study highlights variations in STH preventive practices. Participants from the Omo Nada, Kersa, and Manna districts reported slight improvements in their preventive measures, with average increases of 0.1, 0.09, and 0.12, respectively, compared to those from the O/Beyam district. Although the improvements in preventive practices are modest, they still reflect a positive trend toward adopting measures that could potentially reduce the spread and impact of STH infections.

The differences in knowledge and practices between districts could be attributed to several factors, such as the effectiveness of health education campaigns, accessibility to resources, or cultural practices. It underscores the importance of tailoring public health interventions to the specific needs and contexts of different communities to improve both knowledge and preventive behaviors related to STH. Additionally, understanding the reasons behind the disparities can inform future strategies to enhance education and preventive practices in underperforming districts like O/Beyam.

On the other hand, participants with sufficient hand washing knowledge at critical times were more likely to practice STH prevention measures. This is might be due to individuals with hand washing knowledge more likely to have awareness of the recommended preventive practices of STH, aware of the source of contamination and might have more information on STH.

Regarding hand washing, almost all (95.8%) knew the proper method of hand washing. However, the magnitude of hand washing knowledge (42.5%) and practice (43.9%) at critical times were insufficient. Indeed, the magnitude of adequate practice on frequency of hand washing at critical times agreed with study conducted in Gedeo Zone, Ethiopia 44.9% [73]. But, it is higher than study conducted in Benchmaji, Ethiopia 34.6% [74]. However, it is lower than study conducted in Addis Ababa 74.4% [75], Debark town, Amhara, Ethiopia 52.2% [76] and 87% in Wondogenet, Oromia [77]. The possible differences could be due to differences in socioeconomic status, sample size, study period, study area coverage, access to health information, living standard difference among the study settings and the difference in the tool used for assessing hand washing practice and techniques of assessment. For instance, study done in Addis Ababa was conducted in the country’s capital city, which might help them to better access to health information and awareness, whereas study conducted in Wondogenet district which covered only one kebele as opposed to our case, where several kebeles participated and lower prevalence in some kebeles or districts might have masked higher the prevalence among other kebeles or districts in our study area.

People age from 15 to 35 years had better adequate hand washing practices at critical times compared with age of greater than 45 years. This implies that there was a disparity among different age groups of the community members in washing their hand at critical times frequently. Additionally, participants who had the improved latrine washed their hands more frequently compared with their counterparts. This might be due to the availability of hand washing facilities. Finally, hand washing knowledge at critical times is significantly associated with hand washing practice which is in harmony with the previous studies [76]–[78] and not in other studies [79], [80]. This suggests that practicing proper hand washing requires more than just knowledge; other factors may also be involved, and changing the behavior is a challenging task [81], [82]. The results of the qualitative study also showed that with the exception of children, personal hygiene had improved.

In this study, 55.2% of study participants perceived themselves to be at high risk for STHI. This means people’s belief about the likelihood of acquiring STHI was not sufficient where STHI is endemic. These highlight the necessity of developing health education programs and carrying out communication interventions for social and behavioral changes. Moreover, the average risk perception score varied among the groups of educational status, study district and age category of respondents. This might be as a result of the relationship between educational status and knowledge which can influence risk perception towards STHI. Furthermore, there might be variations in the way the STH control program is implemented in each district and the accessibility of health education, which could affect how differently people perceive risk in each study district. Lastly, our findings showed that there are variations in terms of their risk perception among age groups towards STHI which might be due to difference in their awareness level, perceived consequences and access to health information.

## Conclusions

Even though almost all of the participants are exposed to STH communication opportunities, their level of comprehensive knowledge, perception and preventive practices were 46.7%, 55.2% and 44.4% respectively and it varied among the districts of the zone. Educational status and latrine status were significantly associated with overall knowledge of STH, whereas knowledge specific to hand washing and overall knowledge were predictors of STH preventive practice. Furthermore, washing hands during critical times was moderately improved among the young-aged, knowledgeable on hand washing and having improved latrine.

The findings from this study have important implications for practice, the existing literature, and public health policy considerations on strengthening STH prevention efforts. Specifically, the study highlights the importance of comprehensive knowledge about STH, as well as knowledge about handwashing, as key components of STH prevention strategies. Furthermore, the study found that handwashing practices at critical times were affected by factors like age category and latrine status. This underscores the need to account for relevant demographic and infrastructure-related factors that influence handwashing behaviors when designing interventions. Overall, the findings demonstrate the central role of knowledge in effective STH prevention strategies as it is a necessary condition for the behavior change to occur. At the same time, the study emphasizes the need to account for relevant demographic and infrastructure-related factors that influence handwashing behaviors.

Understanding the level of knowledge, perception and preventive practices can inform development of targeted public health interventions and policies aimed at reducing the prevalence of STHI. The findings can enhance community empowerment by providing accurate information on STH, which can lead to proactive measures to protect individual and community health and well-being. Thus, our findings highlight the need to develop and strengthen context-specific social and behavior change communication interventions which are tailored to local contexts. These interventions should be integrated into STH control programs, including existing opportunities such as MDA campaigns, to enhance and sustain community knowledge and adoption of effective STH prevention measures.

### Strength and limitations of the study

The strengths of this study included the large sample size, community-based approach, and use of mixed methods. However, the study also had some limitations. One limitation was the potential for self-reporting bias, especially regarding preventive practices for STH. Additionally, there was a possibility of recall bias affecting some of the data. On our analysis, Kruskal-Wallis Test can’t determine which specific groups differ from each other and does not provide information about the size. Similarly, Mann-Whitney U Test also does not provide information about the magnitude of the effect. Finally, with qualitative analysis there might be researcher subjectivity and the software lack automated coding and theme generation features, which can make the analysis process more labor-intensive and potentially less consistent.

## Supporting information

S1 Fig The figure summarizing the study design on community’s knowledge, perceptions and preventive practices on STH in Jimma zone, Ethiopia. (DOCX)

S1 Material The Data used in the analysis. (SAV)
