# Supplementary material for: Communities’ knowledge, perceptions and preventive practices on soil-transmitted helminthes in Jimma, Oromia, Ethiopia: Formative mixed study
Source: PLoS Negl Trop Dis. 2024 Sep 20;18(9):e0012483. doi: 10.1371/journal.pntd.0012483 (PMC11414906; doi:10.1371/journal.pntd.0012483)
Supplement: S1 Fig — (DOCX) [file pntd.0012483.s001.docx]

**Supplementary fig 1**

**Fig 1:** The figure summarizing the study design on community’s knowledge, perceptions and preventive practices on STH in Jimma, Ethiopia
